# Long-Term Follow-Up of Resistance-Associated Substitutions in Hepatitis C Virus in Patients in Which Direct Acting Antiviral-Based Therapy Failed

**DOI:** 10.3390/ijms18050962

**Published:** 2017-05-03

**Authors:** Kanako Yoshida, Hoang Hai, Akihiro Tamori, Yuga Teranishi, Ritsuzo Kozuka, Hiroyuki Motoyama, Etsushi Kawamura, Atsushi Hagihara, Sawako Uchida-Kobayashi, Hiroyasu Morikawa, Masaru Enomoto, Yoshiki Murakami, Norifumi Kawada

**Affiliations:** Department of Hepatology, Osaka City University Graduate School of Medicine, Osaka 545-8585, Japan; m2028081@med.osaka-cu.ac.jp (K.Y.); hhai@med.osaka-cu.ac.jp (H.H.); m2012439@med.osaka-cu.ac.jp (Y.T.); m1159834@med.osaka-cu.ac.jp (R.K.); myhr8@med.osaka-cu.ac.jp (H.M.); etsushi-k@med.osaka-cu.ac.jp (E.K.); hagy@med.osaka-cu.ac.jp (A.H.); sawako@med.osaka-cu.ac.jp (S.U.-K.); morikawa-h@med.osaka-cu.ac.jp (H.M.); enomoto-m@med.osaka-cu.ac.jp (M.E.); yoshimurak@med.osaka-cu.ac.jp (Y.M.); kawadanori@med.osaka-cu.ac.jp (N.K.)

**Keywords:** NS5A, RAS, DAA failure, SMV, ASV, DCV

## Abstract

We evaluated the transition of dominant resistance-associated substitutions (RASs) in hepatitis C virus during long-term follow-up after the failure of DAAs (direct acting antivirals)-based therapy. RASs in non-structure (NS)3/4A, NS5A, NS5B, and deletions in NS5A from 20 patients who failed simeprevir/pegylated-interferon/ribavirin (SMV/PEG-IFN/RBV) and 25 patients who failed daclatasvir/asunaprevir (DCV/ASV) treatment were examined by direct sequencing. With respect to SMV/PEG-IFN/RBV treatment, RAS was detected at D168 in NS3/4A but not detected in NS5A and NS5B at treatment failure in 16 of 20 patients. During the median follow-up period of 64 weeks, the RAS at D168 became less dominant in 9 of 16 patients. Among 25 DCV/ASV failures, RASs at D168, L31, and Y93 were found in 57.1%, 72.2%, and 76.9%, respectively. NS5A deletions were detected in 3 of 10 patients treated previously with SMV/PEG-IFN/RBV. The number of RASs in the breakthrough patients exceeded that in relapsers (mean 3.9 vs. 2.7, *p* < 0.05). RAS at D168 in NS3/4A became less dominant in 6 of 15 patients within 80 weeks. Y93H emerged at the time of relapse, then decreased gradually by 99% at 130 weeks post-treatment. Emerged RASs were associated with the clinical course of treatment and could not be detected during longer follow-up.

## 1. Introduction

Chronic hepatitis C (CHC) due to infection with hepatitis C virus (HCV) affects approximately 170 million people worldwide and is the most common cause of chronic liver disease [1]. About 20–30% of HCV-infected individuals eventually develop liver cirrhosis or hepatocellular carcinoma (HCC). The primary aims of anti-HCV therapy for patients with CHC are elimination of the virus and preventing progression to cirrhosis and HCC. Recently, direct-acting antiviral (DAA) regimens with or without interferon (IFN) were approved for anti-HCV therapy and have been evaluated. The protease inhibitors telaprevir, simeprevir (SMV), and vaniprevir were approved in Japan as DAA combination therapy with pegylated (PEG)-IFN and ribavirin (RBV) [2,3,4]. Daclatasvir (DCV) plus asunaprevir (ASV) was initiated as the first IFN-free anti-HCV combination therapy in 2014. Although these DAA therapies achieve a higher sustained viral response (SVR) rate than conventional IFN-based therapy [5,6], a resistant HCV variant emerged in patients who did not achieve an SVR [7,8,9,10]. Clinical data show that resistance-associated substitutions (RASs) matched to the DAA used occurred in patients who failed in DAA therapy [11]. In other words, the DAAs used (with the same resistance profile) may have lost their anti-HCV effect in patients following DAA therapy failure. A follow-up analysis showed that resistant variants after treatment failure were persistently dominant in patients who were administered NS5A inhibitors [9]. The variants resistant to NS3/4A inhibitors progressively disappeared after treatment was terminated [8].

However, the relationship between emerging RAS and the clinical course of DAA therapy has not been evaluated. In addition, few reports are available on the alterations in the emerged RAS after DAA therapy for more than 52 weeks.

In this study, we evaluated the characteristics of HCV RASs in NS3/4A, NS5A, and NS5B in patients who failed SMV/PEG-IFN/RBV, DCV/ASV, or DCV/ASV after SMV/PEG-IFN/RBV therapy.

## 2. Results

### 2.1. Resistance-Associated Substitutions (RASs) in the NS3/4A, NS5A, and NS5B Regions of HCV after SMV/PEG-IFN/RBV Treatment

**Figure 1:**
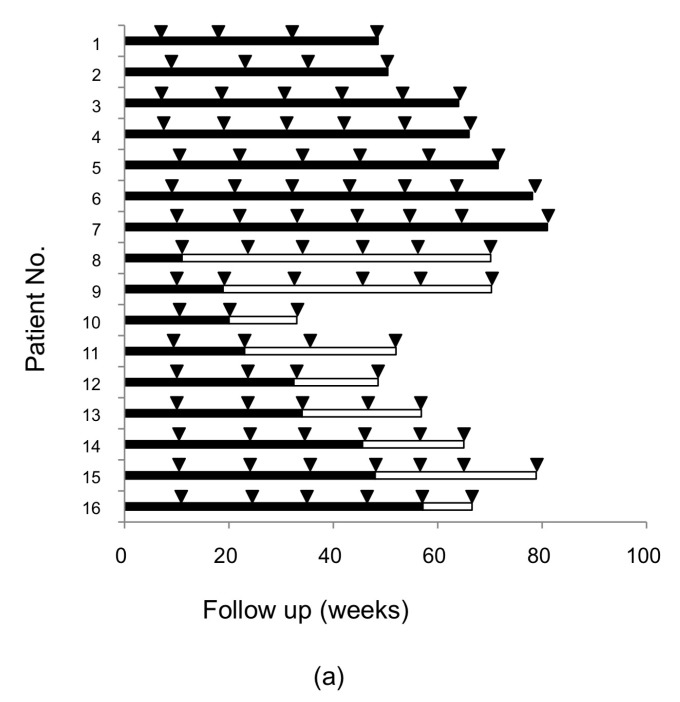

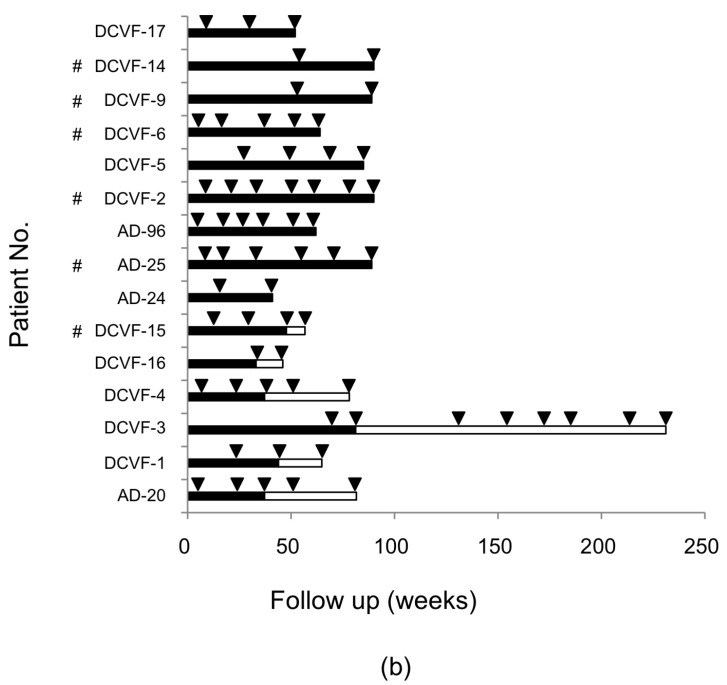
Alteration of D168 resistance-associated substitution (RAS) during follow-up after treatment failure. (**a**) Sixteen patients in simeprevir/pegylated-interferon/ribavirin (SMV/PEG-IFN/RBV) and (**b**) Fifteen patients in daclatasvir/asunaprevir (DCV/ASV) treatments were followed-up D168 RAS. Each line indicates an individual patient; the closed bar indicates a continuous predominant substitution and the open bar indicates a substitution reverting to the wild-type. Arrowheads indicate the point when RAS was determined. #: Patients with prior treatment of SMV/PEG-IFN/RBV.

RASs in the NS3/4A, NS5A, and NS5B regions and deletions in the NS5A region were analyzed in 20 patients who failed SMV/PEG-IFN/RBV treatment. At the baseline, RASs at Q80, D168, and V170 in the NS3/4A region were observed in 5.0% (1/20), 0.0% (0/20), and 25.0% (5/20) of cases, respectively. At treatment failure, RASs at Q80, D168, and V170 were observed to be more prevalent in 15.0% (3/20), 80.0% (16/20), and 30.0% (6/20) of the patients, respectively. At 48 weeks follow-up, those RASs reverted to the wild-type in 33.3% (1/3), 56.3% (9/16), and 0.0% (0/6) of the patients, respectively (Figure 1a). We also compared background characteristics between the two groups and found that the platelet count in patients whose D168 substitution reverted to the wild-type was higher than that in those substitution did not (16.1 × 10^4^/µL vs. 11.9 × 10^4^/µL, *p* < 0.05, Table 1).

In addition, at the baseline, RASs at R30, L31, A92, and Y93 in the NS5A region were observed in 0.0% (0/17), 0.0% (0/17), 5.9% (1/17), and 11.8% (2/17) of cases, respectively. No deletion in NS5A or RAS in NS5B was detected either before or after treatment failure.

### 2.2. RASs in the NS3/4A, NS5A, and NS5B Regions of Hepatitis C Virus (HCV) after Daclatasvir/Asunaprevir (DCV/ASV) Treatment

RASs in the NS3/4A, NS5A, and NS5B regions and deletions in the NS5A region were analyzed in 25 patients who failed DCV/ASV treatment (Table 2). Because limited samples were available at the baseline, NS3/4A RASs at Q80, D168, and V170 were observed in 27.3% (3/11), 36.4% (4/11), 66.7% (6/9), respectively; NS5A RASs at R30, L31, A92, and Y93 were observed in 11.1% (1/9), 5.3% (1/19), 0.0% (0/9), and 31.6% (6/19). At treatment failure, NS3/4A RASs at Q80, D168, and V170 were found in 24.0% (6/25), 76.0% (19/25), 52.0% (13/25), and NS5A RASs at R30, L31, A92, and Y93 were found in 28.0% (7/25), 76.0% (19/25), 8.0% (2/25), and 80.0% (20/25), respectively. Interestingly, P29 or P32 deletions were observed in the NS5A region in 12.0% (3/25) of the patients (GenBank accession numbers: KY969635, KY969636, and KY969637), all of whom had a history of SMV/PEG-IFN/RBV treatment. No RAS was observed at S282 in the NS5B region. Stratified by the presence of a history of SMV treatment, the proportions of breakthrough in the DCV/ASV failure patients differed (breakthrough in 100% (10/10) of patients with a history of SMV treatment vs. 53.3% (8/15) of DAA-naïve patients, *p* < 0.05). The median (range) duration of the SMV and DCV/ASV treatment was 24 (8–32) weeks.

These results showing the emergence of considerable variation in the number of RASs in each patient encouraged us to analyze the association between the number of RASs and the response to the duration of DCV/ASV treatment. Consequently, significantly more RASs were observed in DCV/ASV breakthrough patients than in relapsed patients (mean 3.9 vs. 2.7, *p* < 0.05). About 55.5% (10/18) of the breakthrough patients had a history of SMV/PEG-IFN/RBV treatment. Even when excluding SMV/PEG-IFN/RBV failure, the same tendency was observed (4.0 vs. 2.7, *p* = 0.055). The correlation coefficient between the number of RASs and DCV/ASV duration was 0.19.

### 2.3. Alteration of RASs at D168 in the HCV NS3/4A Region and at Y93 in the NS5A Region in Patients Who Failed DCV/ASV Treatment

Among 25 patients who failed DCV/ASV therapy, fifteen patients were followed for a median of 78 (41–231) weeks. One patient who had participated in a Japanese phase III clinical trial and was treated with DCV/ASV [12] was followed for >231 weeks. The observation periods were 41–90 weeks in the other patients. RASs at Q80, D168 and V170 in NS3/4A were detected in 4, 11, and 6 patients at treatment failure; RAS at D168 in NS3/4A reverted to the wild-type in 6 patients during 33–80 weeks of observation while RAS at Q80 or V170 did not revert at all (Figure 1b). The platelet count was higher in patients whose D168 substitution reverted to the wild-type than in those without the reversion (17.4 × 10^4^/µL vs. 9.2×10^4^/µL, *p* < 0.05). There were also significant differences in the prothrombin time, albumin, and total bilirubin levels at baseline between the two groups (Table 3).

**Figure 2:**
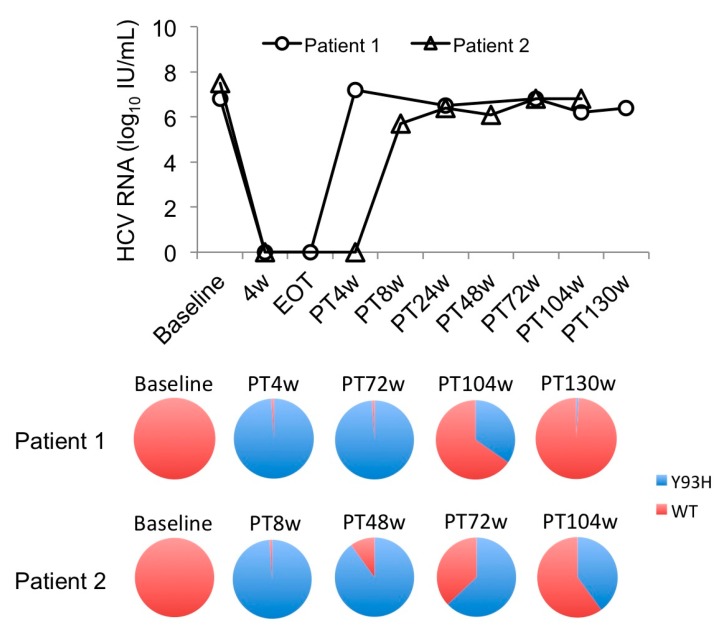
Reversion of the emerged Y93H substitution to the wild-type after DCV/ASV treatment failure. The upper panel shows the viral load of 2 followed patients. The lower panel shows the Y93H RAS signal at baseline and after DCV/ASV therapy. The Y93H RAS emerged at the time of relapse in both patients and gradually returned to the wild-type 130 weeks post-treatment (PT130w) in patient 1 and was about to return to the wild-type in patient 2.

Next, the Y93H substitution was detected in the NS5A region in the 13 patients while the other two patients had a deletion mutation in NS5A. The Y93 RAS was continuously predominant in all patients during the 72 weeks of observation. However, the Y93 RAS reverted or was going to revert to the wild-type 100 weeks post-treatment (PT100w) in two patients. The proportion of Y93H was quantitatively analyzed using a polymerase chain reaction (PCR) invader assay. In patient 1, the proportion was >99% from the time of relapse (PT4w) until 72 weeks, then declined to 34% at 104 weeks, and finally became undetectable (<1%) at 130 weeks after the end of treatment (Figure 2). In patient 2, Y93H emerged (>99%) also at the time of relapse (PT8w), then gradually decreased the RAS signal to 90%, 63%, and 40% at 48, 72, and 104 weeks post-treatment. In addition, RASs at R30, L31 and A92 were detected in 5, 7 and 1 patient, respectively, at treatment failure. The RASs at R30 and A92 reverted to the wild-type in one patient during 24 weeks of observation, while the RAS at L31 did not.

## 3. Discussion

Previous studies have shown that patients who experience treatment failure using an NS3/4A protease inhibitor and/or an NS5A inhibitor had viruses with amino acid substitutions that conferred drug resistance in the NS3/4A protease and NS5A regions, respectively [8,9]. Before the ledipasvir/sofosbuvir combination was approved in Japan in 2015, we used SMV/PEG-IFN/RBV or DCV/ASV to treat HCV genotype 1b. The present study confirms these results. Specifically, a substitution in NS3/4A at D168 emerged at the time of failure in 16 of 20 patients who failed SMV/PEG-IFN/RBV therapy. There was no substitution in the NS5A or NS5B region in SMV/PEG-IFN/RBV failure. Four relapsed patients did not have detectable RAS in NS3/4A after therapy. It is difficult to explain why the RAS did not emerge in these four patients, even after evaluating their clinical background, drug compliance, *IL28B*, and fibrosis stage. RASs with NS3/4A and/or NS5A amino acid substitutions emerged in patients who failed IFN-free DCV/ASV therapy. However, the pattern of amino acid replacements in the emerged RASs was not identical. RASs were detected at D168 in NS3/4A and at L31 and Y93 in NS5A in 10 (56%) of 18 breakthrough patients and in 1 (14%) of 7 relapsers. In addition, the number of amino acid substitutions was significantly higher in breakthrough patients than in relapsed patients (mean 3.9 vs. 2.7, respectively, *p* < 0.05). The replicons containing L31V or Y93H RAS were demonstrated to be 15- and 12-fold, respectively, more resistant than the wild-type HCV 1b replicon. The replicon containing both L31V and Y93H RASs was 5425-fold more resistant [13]. Therefore, HCV with multiple amino acid substitutions may be very resistant to DAAs. This assumption is supported by our finding that the RASs in breakthrough patients had many amino acid substitutions compared with the RASs in relapsed patients.

In this study, we detected unique deletions at P29 and P32 in the NS5A region in patients retreated with DCV/ASV who had failed SMV/PEG-IFN/RBV therapy. NS5A-deleted variants were shown to have extremely high resistance to NS5A inhibitors in vitro [14]. HCV with an NS5A deletion mutation was not detected in DAA-unexposed patients in a global HCV genotype 1 analysis [15], and was absent in our 825 patients examined by direct sequencing (Figure S1). We also failed to detect NS5A amino acid substitutions in 20 patients treated with SMV/PEG-IFN/RBV. Uchida et al. [16] also detected the same NS5A deletions in three of five DCV/ASV failures. Ultra-sequencing analysis did not detect the NS5A deletion in SMV/PEG-IFN/RBV failure [16]. We speculated that previous triple therapy including SMV potentiates the occurrence of rare NS5A deletions during DCV administration, although the precise mechanism needs to be studied.

Our follow up study revealed that the RAS in NS3/4A reverted to the wild-type within 1 year in 9 of 16 SMV/PEG-IFN/RBV failures and in 6 of 15 DCV/ASV failures. This result is consistent with previous observations [8]. We speculate that RASs in NS3/4A have no proliferation advantage in the absence of DAAs, compared to wild-type HCV. However, RAS in NS3/4A predominantly continued in the remaining 13 non-SVR patients 1 year after treatment failure. We observed that continuous RAS was associated with a lower platelet count, lower prothrombin time, higher albumin, and higher total bilirubin in DCV/ASV failures, suggesting that advanced hepatic fibrosis blunts the reversion of a substitution to the wild-type. Conversely, resistant variants to NS5A inhibitors persist for years (maybe forever) after treatment failure, either because variants are naturally more fit or because they are unable to revert to the wild-type virus for genetic reasons [7,9,17]. However, here, we report two cases, for the first time, in whom Y93H resistant variants were going to revert to the wild-type over 100 weeks. In one case, Y93H persisted for 72 weeks after the end of treatment, but the signal strength decreased gradually, and finally returned to the wild-type after 130 weeks. This observation suggests that the RASs of DAA failures should be monitored over the long-term.

This study has several limitations. First, we could not detect <20% of the nucleotide mutations related to amino acid replacement because deep sequencing was not used. Second, relatively few patients were enrolled in this study. Currently, more than 95% of patients with genotype 1b achieved an SVR due to the development of highly efficient inhibitors for NS3/4 A, NS5A and NS5B, and so it has become difficult to collect non-SVR patients treated with DAA combination therapy. Our study included ten patients who had failed SMV/PEG-IFN/RBV therapy and then failed retreatment with DCV/ASV. Consequently, such a DAA treatment strategy is unfavorable. Finally, the observation period for DCV/ASV failures was up to 72 weeks, except for one patient. An observation for more than 2 years from the end of DAA therapy might reveal the gradual transition of emerged RAS to the baseline amino acid sequence.

In conclusion, this study indicates that the pattern of RASs after DAA failure was associated with breakthrough or relapse and with a history of prior DAA, and the emerged RASs in the NS3/4A and/or NS5A regions were not continuously predominant after DAA therapy failure.

## 4. Materials and Methods

### 4.1. Patients

This study protocol complied with the ethical guidelines of the 1975 Declaration of Helsinki (2013 version) and was approved by the Ethics Committee of Osaka City University Graduate School of Medicine (approval nos. 2509 on 13 March 2013, 2677 on 30 October 2013, and 2905 on 1 September 2014). Written informed consent was obtained from all patients.

This study enrolled 45 HCV genotype 1b infected patients who did not achieve an SVR after 24 weeks of SMV/PEG-IFN/RBV or DCV/ASV treatment. The SMV/PEG-IFN/RBV failure involved 16 patients who had a viral relapse defined as undetectable HCV RNA at the end of treatment with detectable HCV RNA during follow-up, and 4 patients who had a viral breakthrough defined as detectable HCV RNA at the end of treatment (Table 4). The SMV/PEG-IFN/RBV regimen was 100 mg of SMV/day combined with PEG-IFNα (1.5 µg/kg per week) and body weight-based RBV (600–1000 mg/day) for 12 weeks, followed by PEG-IFNα and RBV for 12 weeks. All 20 patients were treated at Osaka City University Hospital. No patient overlapped in the two cohorts.

The DCV/ASV regimen was 100 mg of ASV twice daily plus 60 mg of DCV once daily for 24 weeks for 7 patients at Osaka City University Hospital and 18 patients at another hospital. DCV/ASV failure comprised 18 breakthrough patients and seven relapsed patients (Table 4). Liver cirrhosis was diagnosed based on a liver biopsy result or a transient elastography score of 12.5 kPa. To assess liver fibrosis, the FIB-4 index was calculated according to the formula: (age × AST (IU/L))/(platelet (×10^3^/μL) × √AST (IU/L)) [18]. Among the relapsed patients, three had discontinued the treatment after 5 weeks due to adverse events. Ten patients failed to achieve an SVR with SMV/PEG-IFN/RBV followed by DCV/ASV therapy. After the treatments, the non-SVR patients were followed at Osaka City University Hospital every 12–16 weeks, and blood samples were taken during the visits.

### 4.2. RNA Extraction, cDNA Synthesis, and Direct Sequencing of the NS3/4A, NS5A, and NS5B Regions of the HCV Genome

Serum samples were obtained from the patients before treatment, at treatment failure, and during each follow-up visit. RNA was extracted from 140 µL of serum with a commercial kit (QIAamp viral RNA mini kit; QIAGEN, Hilden, Germany). Complementary DNA (cDNA) was synthesized from the RNA using a random primer and a reverse transcriptase kit (Transcriptor First Strand cDNA Synthesis Kit; Roche Diagnostics, Basel, Switzerland).

Template cDNA was amplified by PCR using DNA polymerase (KOD-Plus-Ver.2; TOYOBO, Osaka, Japan) with primer pairs specific for the NS3 region of the HCV genome (NS3-F1 (5′-ACACCGCGGCGTGTGGGGACAT-3′; nucleotides 3295–3316) and NS3-AS2 (5′-GCTCTTGCCGCTGCCAGTGGGA-3′; 4040–4019)) or primer pairs specific for the NS5B region (NS5B-F (5′-GCGTCCAACCAGAGAAAGGA-3′; 8023–8042) and NS5B-R (5′-TGCGCTAAGACCATGGAGTC-3′; 8999–8980)); this region was also amplified by nested PCR using DNA polymerase (KOD-Plus-Ver.2; Toyobo) with primers pairs specific for the NS5A region of the HCV genome (NS5A-F1 (5′-AAGAGGCTCCACCAGTGGAT-3′: 6213-6232) and NS5A-AS1 (5′-CGCCGGAGCGTACCTGTGCA-3′: 6730-6749) as the first primer pair and NS5A-F2 (5′-AATGAGGACTGCTCCACGCC-3′: 6234-6253) and NS5A-AS2 (5′-GTGAAGAATTCGGGGGCCGG-3′: 6690–6709) as the second primer pair). The amplified products were purified with a QIAquick PCR purification kit (QIAGEN) and subjected to sequencing PCR using a BigDye Terminator v3.1 Cycle Sequencing Kit (Applied Biosystems, Foster City, CA, USA) according to the manufacturer’s instructions with the primer pairs NS3-F3 (5′-CAGGGGTGGCGGCTCCTT-3′: 3390–3407) and NS3-AS2, NS5A-F3 and NS5A-AS3, or NS5B-SeqF (5′-TTTACGACGTGGTCTCCACC-3′: 8110–8129) and NS5B-SeqR (5′-ACCTAGTCATAGCCTCCGTGA-3′: 8622–8602). Then, the samples were sequenced on a 3130xl Genetic Analyzer (Applied Biosystems). The sense and anti-sense strands were confirmed in each sequence. A sensitivity level of 20% was assumed.

RASs at amino acids Q80, D168, and V170 in the NS3/4A region, at amino acids R30, L31, A92, and Y93 in the NS5A region, and at amino acid S282 in the NS5B region as well as deletions in the NS5A region that were closely associated with in vitro drug resistance and DAA treatment failure were analyzed.

### 4.3. Quantitative Y93H Assay of the NS5A Region of the HCV Genome

A quantitative analysis of a variant at amino acid 93 in the NS5A region of the HCV genome was performed with the invader assay as described previously [19]. Briefly, template cDNA was amplified with the NS5A-F3 and -AS3 primer pair and probes designed for Y93 of the HCV NS5A gene. Fluorescence values were measured at the end of the incubation/extension step in each cycle using standard real-time PCR. A cross-point was obtained using the fit point method with LightCycler 480 software (Roche Diagnostics, Basel, Switzerland).

### 4.4. Statistical Analysis

The data were analyzed using JMP ver. 9.0 (SAS Institute, Cary, NC, USA). Differences between groups were evaluated by Wilcoxon’s two-sample test for numerical variables or Fisher’s exact test for categorical variables. A two-tailed *p*-value <0.05 was considered significant.

## Figures and Tables

**Table 1:** Comparison of the two groups stratified by the change in predominance of the resistance-associated substitution (RAS) at D168 after simeprevir/pegylated-interferon/ribavirin (SMV/PEG-IFN/RBV) treatment failure.

Parameter	Returned to HCV at Baseline	Continuous Predominant Substitution	*p*-Value
	(*n* = 9)	(*n* = 7)	
Age (years) ^a^	68 (51–74)	65 (52–69)	0.18
Male/female	4/5	3/4	1.00
*IL28B* (rs8099917) TT/TG or GG	1/8	3/4	0.26
Hemoglobin (g/dL) ^a^	13.5 (12.0–15.3)	13.6 (12.3–16.6)	0.49
Platelets (×10^4^/μL) ^a^	16.1 (12.6–23.6)	11.9 (8.3–17.5)	0.03
ALT (IU/L) ^a^	30 (17–73)	60 (16–161)	0.27
γ-GT (IU/L) ^a^	24 (15–81)	43 (17–96)	0.34
HCV-RNA (log IU/mL) ^a^	6.4 (5.6–7.4)	6.7 (5.9–7.3)	0.67
Elastography (kPa)	8.7 (3.1–10.0)	6.8 (5.6–12.1)	0.74
FIB-4 index ^b^	2.7 (2.1–4.0)	2.8 (2.0–4.9)	0.96
Response to SMV/PEG-IFN/RBV treatment (relapse/breakthrough)	8/1	4/3	0.26
Duration of follow up after treatment (week) ^a^	64 (33–78)	66 (36–72)	0.56

^a^ Median (range); ^b^ calculated on age, AST, platelet and ALT. RAS: resistance-associated substitution; SMV/PEG-IFN/RBV: simeprevir/pegylated-interferon/ribavirin.

**Table 2:** Overview of RASs after daclatasvir/asunaprevir (DCV/ASV) treatment.

No.	Background Liver Status	Previous Treatment	Response to DCV + ASV	Duration of DCV + ASV (Weeks)	RASs after DCV/ASV Treatment								
					NS3/4A			NS5A					NS5B
					Q80	D168	V170	R30	L31	A92	Y93	Deletion	S282
AD-149	CH	none	Relapse	12	Q	D	V	H	L	A	H		S
AD-24	Cirrhosis	none	Relapse	24	Q	D	I	R	V	A	H		S
DCVF-18	CH	PR	Relapse	2	Q	E/D	V	R	V	A	Y		S
AD-58	Cirrhosis	PR	Relapse	5	Q	D/E	M	R	L	A	Y		S
DCVF-3	CH	PR	Relapse	24	Q	Y	V	R	M	A	H		S
AD-130	CH	PR	Relapse	2	L	D	I	R	L	A	H		S
DCVF-4	Cirrhosis	PR	Relapse	24	Q	V	I	Q	L	A	H		S
DCVF-7	CH	none	BT	20	Q	D	V	R	I	A	H		S
DCVF-16	CH	none	BT	14	Q	D	V	Q	M	A	H		S
DCVF-17	CH	none	BT	12	Q	E	I	H	F	A	H		S
AD-20	CH	P	BT	23	Q	V	V	R	M	A	H		S
DCVF-12	Cirrhosis	PR	BT	14	Q	E	V	R	M	A	H		S
AD-96	Cirrhosis	PR	BT	19	Q	E	I	R	F	V	H		S
DCVF-1	CH	PR	BT	14	Q	V	I	Q	M	A	H		S
DCVF-5	CH	PR	BT	11	R	E	I	R/Q	M	A	H		S
DCVF-11	Cirrhosis	SMV + PR	BT	10	Q	E	V	R	V	A	H		S
DCVF-15	Cirrhosis	SMV + PR	BT	10	Q	V	V	R	F	A	N		S
DCVF-9	Cirrhosis	SMV + PR	BT	12	Q	V	V	R	M	A	H		S
DCVF-8	CH	SMV + PR	BT	14	Q	V	V	R	V	A	H		S
DCVF-14	Cirrhosis	SMV + PR	BT	14	L/R	E	I	R	V	A	H		S
AD-25	CH	SMV + PR	BT	22	Q/R	E	I	R	V	A	H		S
DCVF-13	Cirrhosis	SMV + PR	BT	8	K	D	I	Q	L/M	K/E/T/A	Y/H		S
DCVF-6	CH	SMV + PR	BT	4	Q	V	V	R	L	A	Y	Delete 29	S
DCVF-2	CH	SMV + PR	BT	12	K	E	I	R	L	A	Y	Delete 32	S
DCVF-10	Cirrhosis	SMV + PR	BT	14	Q	V	I	R	L/V	A	Y	Delete 32	S

P: PEG-IFN; PR: PEG-IFN/RBV; BT: breakthrough. A: alanine; D: aspartic acid; E: glutamic acid; F: phenylalanine; H: histidine; I: isoleucine; K: lysine; L: leucine; M: methionine; Q: glutamine; R: arginine; S: serine; T: threonine; V: tyrosine; Y: tyrosine; The gray color shows substitutions.

**Table 3:** Comparison of the two groups stratified by altered predominance in the D168 RAS after DCV/ASV treatment failure.

Parameter	Reverted to HCV at Baseline	Continuous Predominant Substitution	*p*-Value
	(*n* = 6)	(*n* = 9)	
Age (years) ^a^	68 (41–71)	70 (49–76)	0.29
Male/female	2/4	6/3	0.31
*IL28B* (rs8099917) TT/TG or GG	2/4	4/5	1.00
Hemoglobin (g/dL) ^a^	13.1 (11.5–14.8)	13.3 (11.9–15.5)	0.72
Platelets (× 10^4^/µL) ^a^	16.1 (12.5–26.0)	9.3 (6.6–21.8)	0.03
Prothrombin time (%) ^a^	99 (94–106)	84 (60–97)	0.03
Total bilirubin (IU/L) ^a^	0.5 (0.3–0.5)	0.7 (0.4–1.5)	0.04
ALT (IU/L) ^a^	46 (11–67)	35 (13–85)	0.91
γ-GT (IU/L) ^a^	35 (14–57)	32 (12–96)	0.72
Alb (mg/mL) ^a^	4.2 (3.9-4.4)	3.9 (2.8-4.6)	0.04
HCV-RNA (log IU/mL) ^a^	6.4 (5.9–7.0)	6.1 (5.6–6.8)	0.19
FIB-4 index ^b^	2.6 (1.1-4.2)	4.3 (1.3-10.0)	0.04
Response to DCV/ASV treatment (relapse/breakthrough)	2/4	1/8	0.53
Follow-up duration after DCV/ASV treatment (week) ^a^	72 (46–231)	85 (41–90)	0.64

^a^ Median (range); ^b^ calculated on age, AST, platelets, and ALT.

**Table 4:** Background characteristics of the non-SVR patients.

Parameter	SMV/PEG-IFN/RBV Failure	DCV/ASV Failure
	*n* = 20	*n* = 25
Age (years) ^a^	66 (40–74)	68 (41–78)
Male/female	10/10	16/9
Previous treatment		
None	6 (30%)	5 (20%)
Conventional IFN	14 (70%)	10 (40%)
SMV/PEG-IFN/RBV	0 (0%)	10 (40%)
*IL-28B* (rs8099917)		
TT	5 (25%)	11 (44%)
TG or GG	15 (75%)	14 (56%)
Hemoglobin (g/dL) ^a^	13.8 (12.0–16.6)	13.1 (10.0–15.5)
Platelets (×10^4^/μL) ^a^	15.5 (8.3–25.4)	13.2 (3.9–31.5)
ALT (IU/L) ^a^	36.5 (16–161)	35 (10–85)
γ-GT (IU/L) ^a^	35 (15–96)	38 (12–96)
HCV-RNA (Log IU/mL) ^a^	6.8 (5.6–7.5)	6.1 (4.6–7.0)
Background liver status		
Chronic hepatitis	20 (100%)	11 (44%)
Cirrhosis	0 (0%)	14 (56%)

^a^ Median (range).

## Supplementary Materials

Supplementary materials can be found at www.mdpi.com/1422-0067/18/5/962/s1.

## Abbreviations

RAS	Resistance-associated substitution
DAA	Direct-acting antiviral
SMV/PEG-IFN/RBV	Simeprevir/pegylated-interferon/ribavirin
DCV/ASV	Daclatasvir/asunaprevir
CHC	Chronic hepatitis C
HCV	Hepatitis C virus
HCC	Hepatocellular carcinoma
SVR	Sustained viral response
