# Supplementary figures and images for: Long-Term Follow-Up of Resistance-Associated Substitutions in Hepatitis C Virus in Patients in Which Direct Acting Antiviral-Based Therapy Failed
Source: Int J Mol Sci. 2017 May 3;18(5):962. doi: 10.3390/ijms18050962 (PMC5454875; doi:10.3390/ijms18050962)

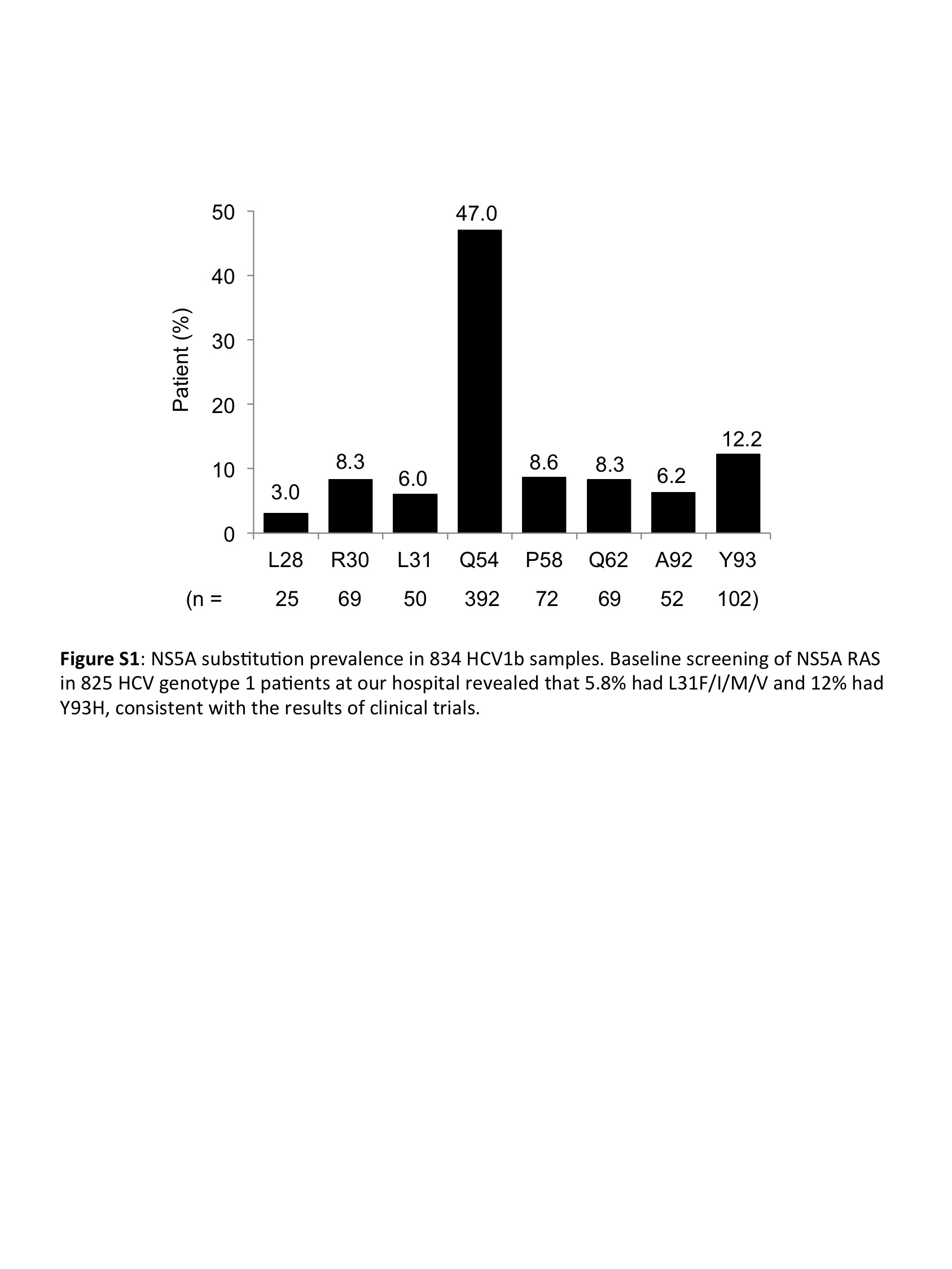

Supplement: Supplementary file 1 [file ijms-18-00962-s001.jpg]
